# Early and mid-term outcomes of minimally invasive mitral valve repair via right mini-thoracotomy: 5-year experience with 129 consecutive patients

**DOI:** 10.1007/s11748-020-01573-2

**Published:** 2021-01-05

**Authors:** Taisuke Nakayama, Yoshitsugu Nakamura, Yuto Yasumoto, Daiki Yoshiyama, Miho Kuroda, Shuhei Nishijima, Yujiro Ito, Ryo Tsuruta, Takaki Hori

**Affiliations:** Department of Cardiovascular Surgery, Chiba-Nishi General Hospital, 107-1 Kanegasaku, Matsudo-shi, Chiba-Ken, Chiba 270-2251 Japan

**Keywords:** Minimally invasive surgery, Mitral valve, Right mini-thoracotomy

## Abstract

**Objectives:**

This study analyzed the experience of a single institution with minimally invasive mitral valve repair (MIMVr) via a right mini-thoracotomy (RT), including short and mid-term morbidity and mortality as surgical outcomes, and rates of reoperation. Late follow-up findings regarding mitral regurgitation (MR) were also assessed.

**Methods:**

Between January 2014 and January 2020, a total of 141 consecutive patients underwent MIMVr for mitral regurgitation at our institution via an RT, with late follow-up results (median 35 ± 15 months) available for 129 (91.4%). Findings regarding surgical approach, complications, reoperations, and late survival were examined. Late echocardiographic results showing recurrence of MR after mitral repair were also noted. Survival, freedom from reoperation, and recurrent MR (grade > 2) were evaluated by Kaplan–Meier analysis.

**Results:**

Mean age was 63.9 ± 14.3 years, mean ejection fraction was 66.9 ± 10.4%, and 2 patients (1.6%) underwent a reoperation. Concomitant procedures included atrial fibrillation ablation (18%), tricuspid valve surgery (16%). None (0%) experienced intraoperative conversion to sternotomy. A learning curve was observed as the number of cases increased. Overall in-hospital mortality and stroke incidence were both 0%. Freedom from recurrent MR (grade > 2) at 1, 3, and 5 years was 99.2, 94.9, and 94.9%, respectively, while freedom from reoperation at 1, 3, and 5 years after mitral valve repair was 98.4, 98.4, and 98.4%, respectively.

**Conclusions:**

Early and mid-term results of MIMVr were satisfactory, with low rates of perioperative morbidity and recurrent MR, as well as reoperation and death. Furthermore, the protocols for patient selection and surgical approach were considered to be appropriate.

**Supplementary Information:**

The online version contains supplementary material available at 10.1007/s11748-020-01573-2.

## Introduction (background)

Minimally invasive mitral valve repair (MIMVr) has been proven to be a feasible alternative to a conventional full sternotomy approach. As compared with conventional surgery, MIMVr has shown excellent early results in terms of morbidity, and providing a shorter hospital stay, and faster recovery and return to normal activities, resulting in reduced use of rehabilitation resources and lower healthcare costs [1–6]. On the other hand, disadvantages reportedly include a longer cross-clamp time and technical demands related to performing a valve repair via a small thoracotomy [2, 6]. These disadvantages may affect long term results of MIMVr. However, little is known regarding the mid-term efficacy of this approach. In 2014, our department started using an MIMVr procedure, with that via a right mini-thoracotomy (RT) now the standard approach for treatment of mitral valve disease. The aim of the present study was to analyze early and mid-term outcomes of 129 consecutive patients who underwent MIMVr via an RT during a 5-year period.

## Materials and methods

### Patient selection and data collection

This retrospective observational study was performed using prospectively collected data of 309 consecutive patients who underwent mitral valve repair procedures between January 2014 and January 2020. All patients were routinely examined using contrast enhanced CT and head and neck magnetic resonance imaging. For preoperative enhanced CT in MIMVr cases, images were obtained with the patient in an exhalation phase with arms down. Patient selection was determined mainly based on the decision of the attending surgeon, though for high-risk patients such as those with impaired left ventricular function, a heart team composed of cardiac surgeons and cardiologists was responsible for the decision. Patients with anatomical and/or physiological problems were evaluated with reference to advice from an appropriate specialist. Notably, cases with a chest wall deformity such as scoliosis or funnel chest were carefully considered. A total of 54 sternotomy procedures were performed, with that modality chosen because of very poor left ventricular ejection fraction (less than 30%), severe peripheral vascular disease [ankle-brachial index (ABI) 0.6 or lower], strong pleural adhesions (history of right lung surgery, radiation therapy, etc.), severe chronic obstructive pulmonary disease (forced expiratory volume during first second < 1 L), active endocarditis, or concomitant coronary artery disease requiring surgical intervention. History of dialysis or impaired renal function was not considered to be a contraindication for MIMVr. Indications for treating liver cirrhosis patients with MIMVr were the same as used for cases of normal cardiac surgery with a full sternotomy procedure.

MIMVr with robotic assistance using the da Vinci system (Intuitive Surgical Inc, Mountain View, CA) was instituted at our institution in June 2018, thus from that time patients were treated with that system and they were excluded from this study. Of 256 candidates for MIMVr via an RT, 255 (99.6%) underwent an effective repair procedure, while 1 (0.4%) required replacement after the repair attempt because of severe annulus and leaflet calcification. After excluding 92 who underwent MIMVr with robotic assistance with the da Vinci system and 22 with concomitant aortic valve replacement, 141 patients who underwent MIMVr via an RT approach were enrolled, of whom 129 (91%) also had late follow-up (longer than 6-month) results available.

The main outcomes investigated were early and late mortality, perioperative complications, and freedom from mitral regurgitation (MR) recurrence and reoperation. Early mortality was defined as death from any cause occurring within 30 days of the operation or before hospital discharge. Stroke was defined as any new focal or global neurological deficit lasting more than 24 h, and was confirmed whenever possible by neurologist and neuro-radiologist examinations. Postoperative stroke was diagnosed when evidence was found of a new neurologic deficit, with the presence of morphologic substrate confirmed by computed tomography or magnetic resonance imaging. Respiratory insufficiency included ventilation failure, and performance of reintubation or a tracheostomy.

All pre- and postoperative transthoracic echocardiography results were obtained using a quantitative method. In cases with more than moderate MR, a qualitative method was added and performed by trained cardiac echography technicians and cardiologists. All patients were examined from 1 to 4 weeks, and again 12 and 24 weeks postoperatively, then were contacted thereafter for follow-up data. All patients in the late phase underwent follow-up examinations every 24 weeks. The median follow-up time was 35 ± 15 months (interquartile range 9–69 months) and follow-up data were 91.4% complete. The severity of MR was graded based on recommendations from the European Society of Cardiology and European Association for Cardio-Thoracic Surgery. Recurrent MR was defined as moderate or severe using a four-point grading system (trivial, 1; mild, 2; moderate, 3; severe, 4).

### Surgical technique

Following general anesthesia with differential lung ventilation, the patient was placed in a 20° left lateral position with a pillow under the left thorax. A cardiopulmonary bypass (CPB) was instituted via the femoral arterial or axillary artery and femoral venous cannulation. We selected the femoral artery for retrograde perfusion and the axillary artery for antegrade perfusion. Axillary cannulation was selected if any of the following criteria were satisfied in the aorta or an iliac artery at any point: (1) thickness of the thrombosis is > 3 mm; (2) thrombosis is more than one-third of total circumference; and (3) calcification present in the total circumference. Femoral cannulation was selected in the remaining patients.

**Fig. 1:**
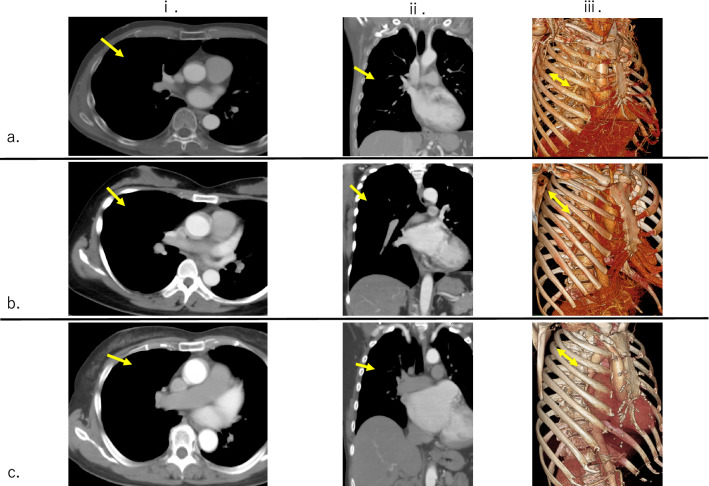
Approach point for right lateral mini-thoracotomy (representative images) (i. axial view, ii. coronal view, iii. 3D image and intercostal approach). **a** Normal patient. The incision was placed in the 4th intercostal space, 2–3 cm lateral from the nipple in males and in the sub-mammary crease in females. **b** Narrow chest patient (sternum-vertebral body length 72 mm). The incision was set more to the lateral side of the anterior axillary line. **c** Obese patient. The incision line was considered to be the 3rd intercostal space

The tip of the venous cannula was positioned in the right atrium under transesophageal echocardiographic (TEE) guidance. A right lateral mini-thoracotomy, 5–6 cm in length, was performed in the 4th intercostal space, with the incision placed 2–3 cm lateral to the nipple in males and in the sub-mammary crease in females (Fig. 1a). For patients with a narrow chest, such as a funnel chest, the incision was placed more to the lateral side of the anterior axillary line (Fig. 1b). Especially for funnel chest cases, double venous cannulas are recommended for positioning the SVC and IVC to secure the surgical field of view. On the other hand, for obese patients in whom the mitral annulus could be compressed by the elevated diaphragm and the heart tended to be in a lying position, the incision was placed more to the cranial side and the approach side was considered to be the 3rd intercostal space (Fig. 1c). Using a small thoracic and soft tissue retractor, a transthoracic Cygnet aortic cross-clamp (Vitalitec Inc, Plymouth, MA, USA) was inserted through the same 4th intercostal space. Antegrade cold blood cardioplegia (6 °C) was administered directly into the aortic root. The first dose included a potassium concentration of 10 mEq/L with flow at 400 mL/min for a concentration of 15 mL/kg, then repeated doses every 40 min including a potassium concentration of 5 mEq/L with flow at 400 mL/min for the same dose given and then repeated within 40 min if necessary. Throughout the procedure, the surgical field was flooded with carbon dioxide through the soft tissue retractor. The left atrium was opened posterior to Sondergaard’s groove and a left atrial retractor was used to expose the mitral valve. Standard mitral valve repair techniques were employed [7]. Deairing was performed by filling the left atrium with saline during closure and via the cardioplegia puncture site on the ascending aorta. In the setting of degenerative mitral valve disease, a mitral valve repair was the first approach attempted for a degenerative mitral valve, while mitral valve replacement was commonly used in cases with severe annulus and/or leaflet calcification.

All patients underwent accurate intraoperative transesophageal echocardiogram examinations performed by an anesthesiologist licensed by the Japanese Board of Perioperative Transesophageal Echocardiography (JB-POT) before and after weaning from the cardiopulmonary bypass machine. MR assessment was performed with a quantitative method. The policy of our institution states that in patients for whom a repair is attempted, the mitral valve should be replaced if (a) some degree of mitral regurgitation is shown in hydrostatic saline test results after several attempts, (b) the coaptation surface is not adequate to guarantee long durability, and (c) intraoperative echo findings show no more than mild MR.

### Statistical analysis

This study was conducted following approval from the Institutional Review Board of Chiba-Nishi General Hospital, with a specific waiver granted for need of individual patient consent. Continuous data are expressed as mean ± standard deviation or median with interquartile range (IQR), and categorical data as percentages. Learning curves and trend lines were created using a two-dimensional scatter plots to show approximate curves for changes in operation, CPB, and aortic cross-clamp (ACC) times. Survival and time-to-event analyses for rates of reoperation, MACE events, and recurrent mitral regurgitation were assessed using the Kaplan–Meier method. The grade of residual or recurrent MR in each patient was compared with baseline preoperative measurements using Student’s *t* test for paired samples. All reported *P* values are two-sided and those < 0.05 were considered to indicate statistical significance. All statistical analyses of recorded data were performed using the Excel statistical software package (Ekuseru-Toukei 2010; Social Survey Research Information Co., Ltd., Tokyo, Japan).

## Results

Patient baseline characteristics and operative procedural details are shown in Table 1. The mean age was 63.9 ± 14.3 years and 47 (36%) were female, while 2 (1.6%) had previously undergone a cardiac operation. Mean preoperative left ventricular ejection fraction was 66.9 ± 10.4% and preoperative chronic atrial fibrillation was noted in 27 (21%). All patients had an MR grade > 3 + . Patients with moderate-severe MR had been hospitalized in the past with heart failure. The most predominant pathology was degenerative disease (*n* = 101, 78%), followed by functional MR including atrial MR (*n* = 14, 11%), endocarditis (*n* = 12, 9%), and rheumatic disease (*n* = 2, 2%). The complexity score for degenerative MR was assessed as previously reported [8] (Appendix Table 5). Of the present study population, 83 (70%) were assigned to the Intermediate and 13 (11%) to the Complex group.

**Table 1:** Preoperative clinical characteristics

Variables	*N* = 129
Mean age, years ± SD	63.9 ± 14.3
Female, *n* (%)	47 (36)
Comorbidities	
Hypertension, *n* (%)	66 (51)
Congestive heart failure, *n* (%)	36 (28)
NYHA class III or IV	32 (25)
Diabetes (insulin user), *n* (%)	9 (7)
COPD, *n* (%)	4 (3)
Atrial fibrillation, *n* (%)	27 (21)
Chronic renal failure, *n* (%)	14 (11)
Dialysis-dependent	0
Previous cardiac operation, *n* (%)	2 (1.6)
Euroscore II, %, mean ± SD	2.1 ± 2.4
Echocardiographic data	
LVEDD, mm, mean ± SD	54.7 ± 5.9
LVESD, mm, mean ± SD	33.9 ± 6.0
LVEF, %, mean ± SD	66.9 ± 10.4
LVEF < 45%, *n* (%)	8 (6.2)
Pulmonary hypertension, *n* (> 45 mmHg)	16 (12)
MR grade	
Moderate-to-severe, *n* (%)	12 (9)
Severe, *n* (%)	117 (91)
Etiology of MR	
Degenerative, *n* (%)	101 (78)
Barlow’s disease	3
Rheumatic, *n* (%)	2 (2)
Infective endocarditis, *n* (%)	12 (9)
Functional, *n* (%)	14 (11)
MV prolapse involved	
Anterior leaflet, *n* (%)	17 (13)
Posterior leaflet, *n* (%)	59 (46)
Bi-leaflet, *n* (%)	39 (30)
Complexity score for degenerative mitral valve	
Simple (score: 1), *n* (%)	22 (19)
Intermediate (score: 2–4), *n* (%)	83 (70)
Complex (score: > 5), *n* (%)	13 (11)

*COPD* chronic obstructive pulmonary disease, *LVDd* left ventricular dimension diastole, *LVDs* left ventricular dimension systole, *LVEF* left ventricular ejection fraction

Operation procedure details are presented in Table 2. The mean operative time was 219 ± 42 min, while ACC and CPB times were 113 ± 35 and 144 ± 36 min, respectively, for the MIMVr. One patient developed moderate-to-severe residual MR during weaning of CPB, thus a second cross-clamping was performed and an additional responsible lesion repaired, followed by weaning with no regurgitation. Concomitant procedures were performed in 57 patients (44%), including atrial fibrillation (AF) ablation in 23 (18%), tricuspid valve surgery in 20 (16%), patent foramen ovale/atrial septal defect closure in 5 (4%), and oversewing or use of a closed devise for the left atrial appendage in 9 (7%). None had an intraoperative conversion to sternotomy.

**Table 2:** Procedural details

Surgical details	*N* = 129
Variates	
Operative time, min, mean ± SD	219 ± 42
Cardiopulmonary bypass time, min, mean ± SD	144 ± 36
Cross-clamp time, min, mean ± SD	113 ± 35
Second aortic cross-clamp, *n* (%)	1 (0.7)
Conversion to sternotomy, *n* (%)	0 (0)
Arterial cannulation: femoral/Rt. axillary artery, *n* (%)/*n* (%)	98 (76)/31 (24)
Venous cannulation: femoral/femoral + SVC, *n* (%)/*n* (%)	115 (89)/14 (11)
MV procedures	
Annuloplasty, *n* (%)	127 (98)
Median ring size, mm (IQR)	28.9 (28–30)
Complete ring, *n* (%)	40 (31)
Posterior band, *n* (%)	87 (69)
Quadrangular/triangular resection, *n* (%)	32 (25)
Folding plasty, *n* (%)	52 (40)
Edge-to-edge repair, *n* (%)	26 (20)
Neochorde placement, *n* (%)	56 (43)
Auto-pericardium augmentation, *n* (%)	1 (0.7)
Concomitant procedures	
Tricuspid valve annuloplasty, *n* (%)	20 (16)
Atrial fibrillation surgery, *n* (%)	23 (18)
ASD closure, *n* (%)	5 (4)
LAA closure, *n* (%)	9 (7)

*IQR* interquartile range

**Fig. 2:**
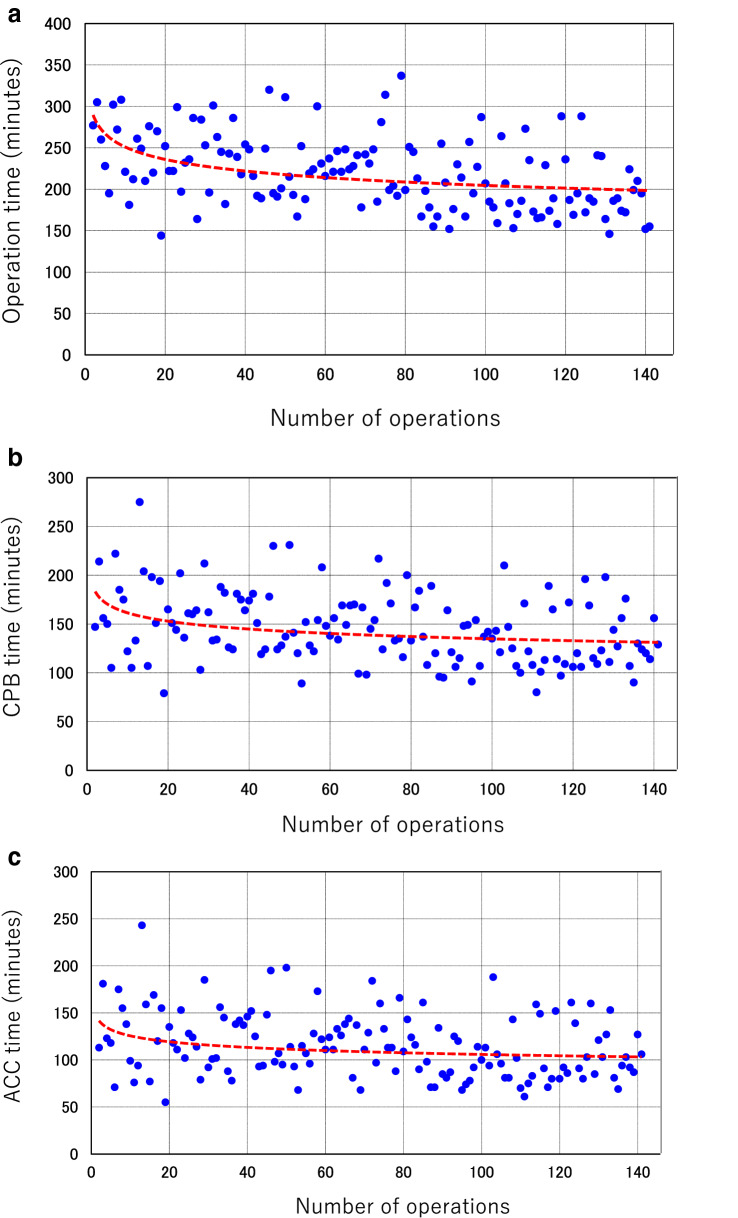
Learning curves and trend lines created by two-dimensional scatter plot and approximate curve. **a** Operative time. **b** CPB time. **c** ACC time

Operation, CPB, and ACC times for the 141 MIMVr cases that underwent continuation, including those that could not be followed for more than 6 months, are shown in Table 3a. The results indicated a significant learning curve for the surgical process (Fig. 2), including operative time [Phase 1 (Case 1–50) 241 ± 42.5 min, Phase 2 (Case 51–100) 220 ± 39.9 min, Phase 3 (Case 101–141) 195 ± 37.3 min; Phase 1 vs. Phase 2: *P* = 0.015, Phase 2 vs. Phase 3: *P* = 0.003] (Fig. 2a). CPB and ACC times were significantly different only between phase 1 and 2, then showed a tendency to be shorter in the most recent cases [CPB: Phase 1, 160 ± 38.6 min; Phase 2, 141.9 ± 30.6 min; Phase 3, 132 ± 31.8 min; Phase 1 vs. Phase 2: *P* = 0.009, Phase 2 vs. Phase 3: *P* = 0.103] (Fig. 2b). [ACC: Phase 1, 127 ± 37.4 min; Phase 2, 113 ± 28.4 min; Phase 3, 105.3 ± 29.7 min; Phase 1 vs. Phase 2: *P* = 0.038, Phase 2 vs. Phase 3: *P* = 0.225] (Fig. 2c). In those that underwent an extended operation, problems encountered included venous cannulation from the lower limbs, such as guide-wire involvement with minor vessels or venous injury, which mainly occurred during Phase 1.

**Table 3:** (a) Transitions of operative data and (b) transitions of mitral valve repair technique and operative data

	Phase 1 case 1–50	Phase 2 case 51–100	Phase 3 case 101–141	P value (Phase 1 vs Phase 2)	P value (Phase 2 vs Phase 3)
Operation time, min, mean ± SD	241 ± 42.5	220 ± 39.9	195 ± 37.3	0.015	0.003
CPB time, min, mean ± SD	160 ± 38.6	141.9 ± 30.6	132 ± 31.8	0.009	0.103
ACC time, min, mean ± SD	127 ± 37.4	113 ± 28.4	105.3 ± 29.7	0.038	0.225

	Phase 1 case 1–50	Phase 2 case 51–100	Phase 3 case 101–141
Quadrangular/triangular resection, *n* (%)	14 (28)	10 (20)	22 (54)
Folding plasty, *n* (%)	20 (40)	29 (58)	19 (46)
Edge to edge repair, *n* (%)	11 (22)	12 (24)	11 (27)
Neochorde placement, *n* (%)	22 (44)	21 (42)	13 (32)

Among patients who underwent a mitral valve procedure, the repair techniques included a ring annuloplasty in 127 (98%) with a median ring size of 28.9 mm (IQR 28–30 mm), which included 31% who received a complete mitral ring and 69% a partial ring. Quadrangular or triangular leaflet resection was performed in 32 (25%), folding plasty in 52 (40%), and polytetrafluoroethylene neo-chordae placement in 56 (43%) cases. Repair technique transitions are listed in Table 3b. During Phase 1, neo-chordae placement or a folding plasty was often required, while choice of a resection suture technique was increased during Phase 3.

### Early outcomes

Operative results are presented in Table 4. Rates of postoperative mortality and morbidity were low. There were no operative deaths (< 30 days or same hospital admission), with the median hospital stay 8 days (IQR 7–11) and ICU stay 2 days (IQR 2–3). A blood transfusion was required during hospitalization in 25 patients (19.4%). New-onset atrial fibrillation occurred in 20 (16%) of the 129 patients admitted for sinus rhythm. Reoperation for bleeding was performed in 2 (1.6%). There were no cerebral vascular accident incidents nor cases of acute renal failure requiring dialysis. 2 patients (1.6%) had pulmonary complications and a permanent pacemaker for complete heart block was required in an additional 2 (1.6%). Following the repair, residual MR at discharge was very low [no MR, *n* = 73 (56.6%); trivial, *n* = 54 (41.8%); mild, *n* = 2 (1.6%); moderate or severe MR, none].

**Table 4:** Early outcomes

Variables	*N* = 129
In-hospital mortality, *n* (%)	0
Intensive care unit stay, days (median, IQR)	2 (2–3)
Hospital stay, days (median, IQR)	8 (7–11)
Duration of ventilation, h (median ± S.D.)	9.1 ± 5.4
Blood transfusion (intra or post op), *n* (%)	25 (19.4)
Low-cardiac output syndrome, *n* (%)	0
Re-exploration, *n* (%)	2 (1.6)
Respiratory insufficiency, *n* (%)	2 (1.6)
Gastrointestinal bleeding, *n* (%)	0
Temporary renal replacement therapy, *n* (%)	0
Cerebral vascular accident, *n* (%)	0
Wound complication, *n* (%)	2 (1.6)
Complete atrioventricular block, *n* (%)	2 (1.6)
New-onset postoperative atrial fibrillation, *n* (%)	20 (16)
Post-operative mitral regurgitation grade, *n* (%)	
None	73 (56.6)
Trivial	54 (41.8)
Mild	2 (1.6)
Moderate or severe	0

### Midterm outcomes

Nine months was used to define the minimum mid-term follow-up period and clinical follow-up was achieved in 129 patients (91.4%), with a mean period of 35 months (range 9–69 months).

### Survival

**Fig. 3:**
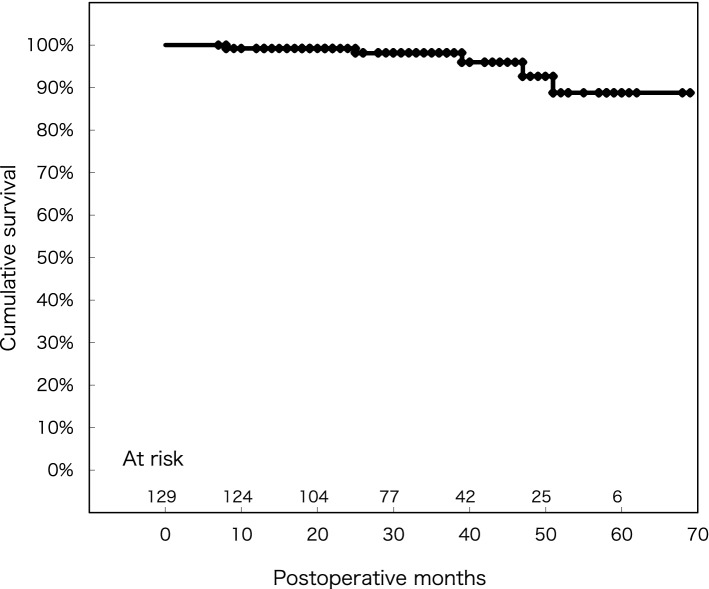
Freedom from late death for any reason

Overall, the 1-, 3-, and 5-year survival rates were 99.2 ± 0.01%, 98.1 ± 0.01%, and 88.8 ± 0.05%, respectively (Fig. 3). A total of five patients (3.9%) died during the follow-up period, though the cause of death could only be determined in 3, none cardiac related (pneumonia, 7 months; gastrointestinal bleeding, 25 months; lung cancer, 28 months; unknown, 8 and 18 months).

### MACE

**Fig. 4:**
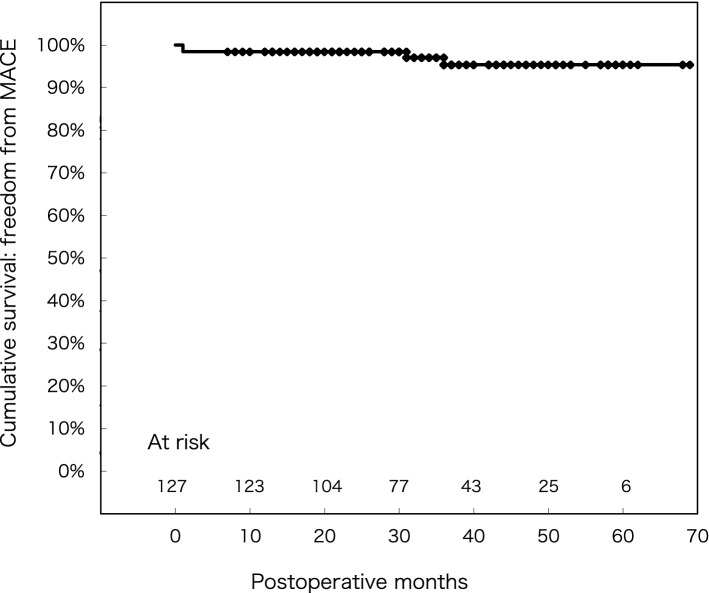
Freedom from MACE

Following mitral valve repair, rates for freedom from MACE events after 1, 3, and 5 years were 100%, 98.9 ± 0.01%, and 95.6 ± 0.03%, respectively (Fig. 4).

### Recurrent MR

**Fig. 5:**
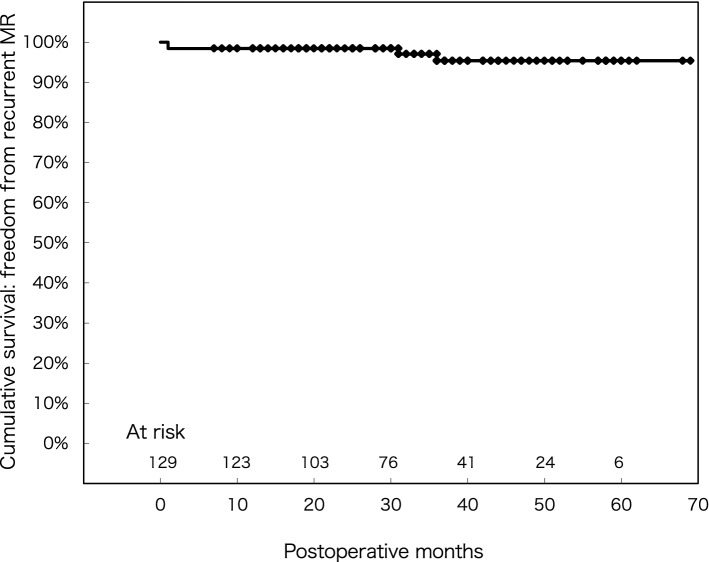
Freedom from recurrent (grade > 2) MR in late follow-up period

For patients who underwent mitral valve repair, freedom from recurrent MR (grade > 2) after 1, 3, and 5 years was 99.2 ± 0.01%, 94.9 ± 0.03%, 94.9 ± 0.03%, respectively (Fig. 5).

### Reoperation

**Fig. 6:**
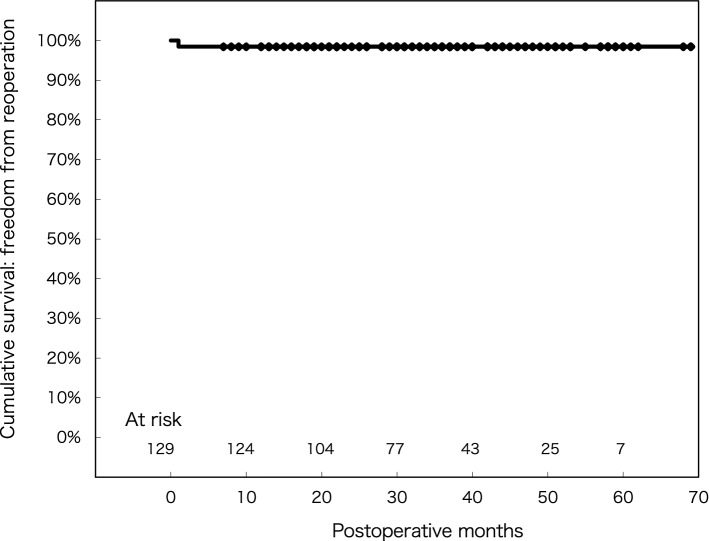
Freedom from reoperation for mitral valve

Freedom from reoperation at 1, 3, and 5 years after mitral valve repair was 98.4 ± 0.01%, 98.4 ± 0.01%, and 98.4 ± 0.01%, respectively (Fig. 6). Two patients underwent a reoperation, 1 for a failed repair the next day of the first MIMVr and 1 for hemolytic anemia more than 1 month after the initial procedure, with the mitral valve successfully replaced under a sternotomy in both.

## Discussion

This study investigated early and mid-term outcomes of MIMVr via an RT performed at our hospital. The present analysis showed that an MIMVr procedure is safe and associated with excellent postoperative outcomes, while our findings also revealed acceptable procedural times, though with a significant learning curve, low levels of residual MR, shorter hospital stays, and excellent long-term results. Specifically, the rates of conversion to a sternotomy and overall in-hospital mortality were both nearly 0%, lower than the recent mortality rate reported in the Society of Thoracic Surgeons database [9]. The initial short-term results with MIMVr have been encouraging, with the present findings suggesting decreased perioperative morbidity. In particular, there were no cerebral vascular accident incidents, likely because we analyzed preoperative contrast CT results to detect the aorta condition, i.e., arteriosclerotic disease or parietal thrombus (see surgical technique subsection above), so as to avoid retrograde perfusion via femoral artery cannulation for CPB. When significant arteriosclerotic disease was found in the entire aorta and iliac artery, we employed right axillary artery cannulation to establish antegrade perfusion for CPB.

In our study, operative time was significantly shorter in the later cases and a significant learning curve was noted. Patient selection and surgical approach were found to be important considerations, especially for the early cases, as operation time tended to be longer, making selection of qualified patients very important. Furthermore, the repair technique showed a transition. We tended to choose a method that did not leave an excessive amount of the mitral leaflet remaining over the latter half, thus a folding plasty was only used for cases with very small sub-lesions.

In contrast to early good results and quick recovery following MIMVr noted in prior reports [10–12], there are concerns regarding its long-term efficacy due to such disadvantages as longer cross-clamp time and technical demands related to performing a valve repair via a small thoracotomy. However, efficacy over a long period has not been adequately elucidated. We found two reports including long-term follow up after MIMVr from high-volume centers. The longest functional and echocardiographic follow-up study was conducted by Glauber and colleagues of MIMVr performed via an RT in 1,137 patients between 2003 and 2013 (mean age 61 ± 13 years, incision length 5–7 cm, in-hospital mortality 1.1%, neurological complications 1.8%, unsuccessful repair rate 6.0%) [13]. Although their unsuccess repair rate was high, their long-term durability of repair was excellent. They reported that 10-year freedom from reoperation was 94%, while freedom from recurrent MR (grade > 2) was noted in 87% and actuarial survival at 10 years was 88%. In 2008, Seeburger et al. reported early and long-term results in a review of a series of 1,339 patients who underwent MIMVr via an RT (mean age 60 ± 13 years, incision length 5–6 cm) [14]. Compared to contemporary reports [10–12], their early results relatively worse (30-day mortality 2.4%, neurological complications 3.1%); however, they found freedom from reoperation in 96.3% of the cases and an overall survival rate of 82.6% at 5-year follow-up examinations. In the present cohort (mean age 64 ± 14 years, mean incision length 5–6 cm), 5-year survival, and freedom from a mitral valve-related reoperation and recurrent MR (grade > 2) showed favorable rates of 88%, 98%, and 95%, respectively. Furthermore, more than 100 of our patients were available for analysis after the mid-term follow-up examination (Figs. 3, 5, 6), thus we are confident regarding the validity of these findings. Confirming the above-mentioned reports, our results indicate that MIMVr is a promising durable procedure.

Unfortunately, we were unable to obtain a conventional sternotomy group control for the current study. Our strategy consists in selecting MIMVr whenever possible, since MIMVr has been our procedure of first line since 2014. Therefore, there is critical selection bias between MIMVr and conventional sternotomy group from our institution. Thus, we sought to compare the present results with the those of conventional full sternotomy mitral valve surgery reported in literature. Freedom from reoperation and survival are important issues to be taken into account when making such comparisons between studies. David et al. reported a series of 701 patients who underwent mitral valve repair for MR due to myxomatous disease using a conventional approach between 1981 and 2001 [15]. Freedom from reoperation at 12 years was 96% for isolated posterior leaflet prolapse, 88% for anterior leaflet prolapse, and 94% for bileaflet prolapse, while overall survival at 12 years was 75%, with no difference regarding the extent of leaflet prolapse. In a report that analyzed mitral valve surgery procedures performed at a single institution, Aubrey et al. presented results of 1071 adults who underwent minimally invasive MV repair and compared those to results of 1601 who underwent sternotomy mitral valve repair over a 12-year period [16]. In isolated valve repair cases, 8-year freedom from reoperation was 91 ± 2% for sternotomy and 95 ± 1% for minimally invasive (*P* = 0.24) procedures, while 8-year freedom from reoperation or severe recurrent insufficiency was 90 ± 2% and 93 ± 1%, respectively (*P* = 0.30). As noted above, our late results in terms of freedom from reoperation or recurrent significant mitral insufficiency are nearly identical to those achieved with the standard sternotomy approach. Furthermore, the excellent late results may support early performance of minimally invasive valve repair for patients with severe mitral insufficiency, prior to development of symptoms or left ventricular dysfunction.

This study has some limitations. The review was retrospective and lacked a control group for appropriate comparisons. Furthermore, inability to determine late cause of death in the majority of patients or perform late echocardiographic assessment in all of the cases are additional weaknesses. In addition, it was not possible to compare this series with a control group, since MIMVr has been our standard approach since 2014 and patients typically demand less invasive procedures. Finally, even though this was a follow-up study conducted over 5 years, the number of patients is small and at the 60-month follow-up examination, the number at risk was only 6. In the future, a well-designed study with an appropriate sample size will be required to validate the advantages of MIMVr.

## Conclusion

Early and mid-term results of MIMVr were satisfactory, with low rates of perioperative morbidity and recurrent mitral regurgitation, as well as reoperation and death. We believe that MIMVr is an attractive alternative to conventional mitral valve repair.

## Supplementary Information

Below is the link to the electronic supplementary material.

Supplementary file1 (PPTX 43 KB)
