# Supplementary material for: Early and mid-term outcomes of minimally invasive mitral valve repair via right mini-thoracotomy: 5-year experience with 129 consecutive patients
Source: Gen Thorac Cardiovasc Surg. 2021 Jan 5;69(8):1174–84. doi: 10.1007/s11748-020-01573-2 (PMC8282559; doi:10.1007/s11748-020-01573-2)
Supplement: Supplementary file 1 — Supplementary file1 (PPTX 43 KB) [file 11748_2020_1573_MOESM1_ESM.pptx]

## Slide 1
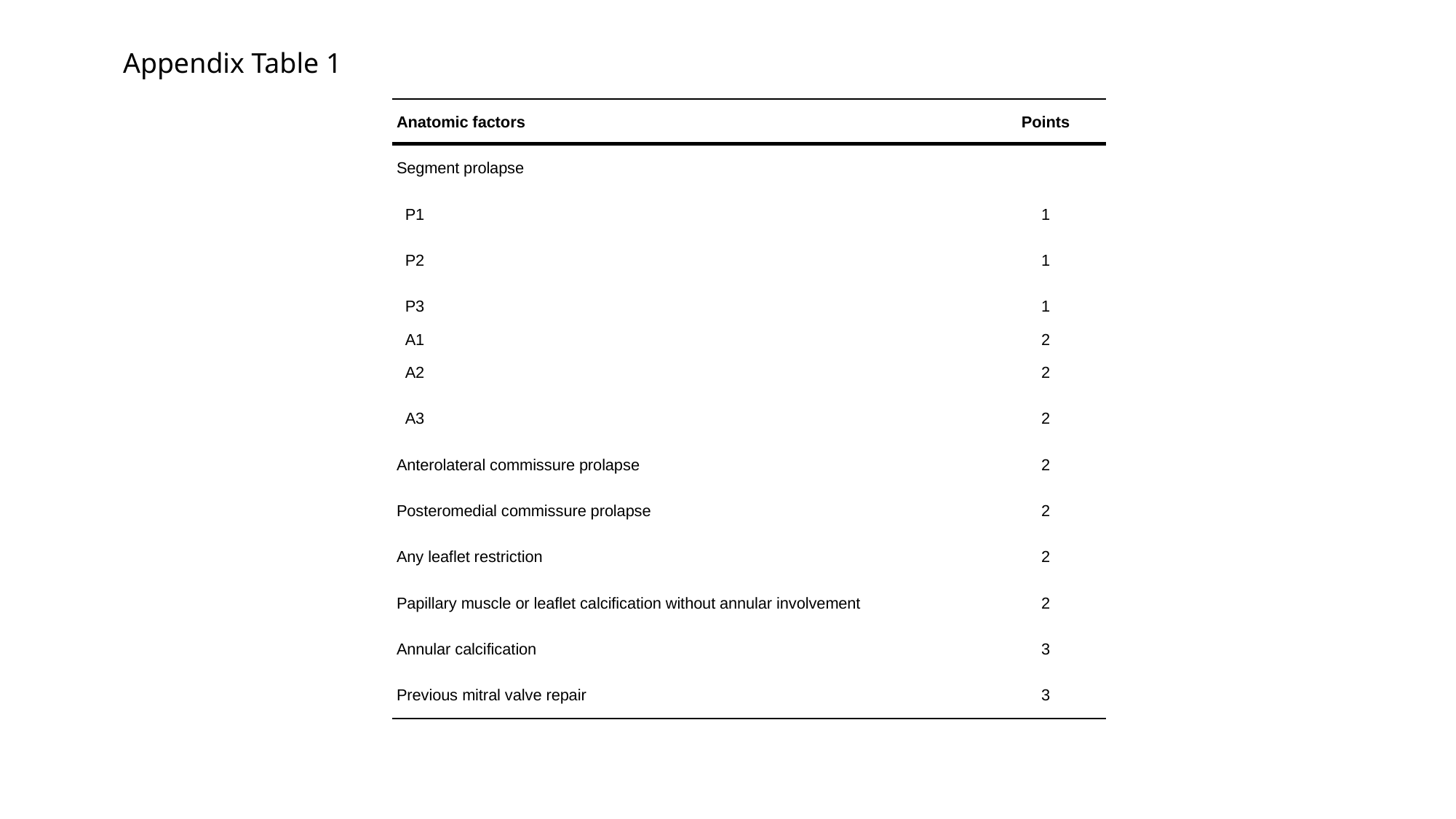

Appendix Table 1
| Anatomic factors | Points |
| --- | --- |
| Segment prolapse | |
| P1 | 1 |
| P2 | 1 |
| P3 | 1 |
| A1 | 2 |
| A2 | 2 |
| A3 | 2 |
| Anterolateral commissure prolapse | 2 |
| Posteromedial commissure prolapse | 2 |
| Any leaflet restriction | 2 |
| Papillary muscle or leaflet calcification without annular involvement | 2 |
| Annular calcification | 3 |
| Previous mitral valve repair | 3 |
